# Experiences of community caregivers in the assessment of malnutrition using mid-upper arm circumference measurement in children under 5 years old

**DOI:** 10.4102/phcfm.v10i1.1743

**Published:** 2018-08-30

**Authors:** Gabisile P. Ndlovu, Dudu G. Sokhela, Maureen N. Sibiya

**Affiliations:** 1 Health Unit, eThekwini Municipality, South Africa; 2 Department of Nursing, Durban University of Technology, South Africa

## Abstract

**Background:**

Malnutrition is a major public health challenge in developing countries. It has been identified as an important cause of child mortality and morbidity and leads to inadequate physical and cognitive development in children. The South African government implemented a strategy for malnutrition assessment in children under 5 years by community caregivers (CCGs), who would then refer children at risk or those having developed malnutrition to primary health care clinics. Irrespective of this strategy, children still present at clinics with severe malnutrition.

**Aim:**

The aim of the study was to explore and describe the experiences of community caregivers with the assessment of malnutrition in children under 5 years old.

**Setting:**

The study was conducted in North Area six of eThekwini district in the province of KwaZulu-Natal.

**Methods:**

A qualitative, exploratory descriptive approach was used to collect data from 13 purposively selected CCGs. Content analysis was used to analyse data.

**Results:**

The majority of participants were dissatisfied with the training, as it was conducted in a language in which they were not proficient. They reported a lack of support and supervision in their performance such that mid-upper arm circumference was non-prioritised. They were dissatisfied with work overload not matched by remuneration and they worked under unsafe conditions.

**Conclusion:**

Effective training of CCGs needs to be conducted in the language that they understand to combat malnutrition in children under 5 years. CCGs have multiple roles and may need to prioritise their work; this is not easy and requires specific guidance from skilled health professionals.

## Introduction

Wasting in children under 5 years old is associated with socio-economic, maternal and child factors in rural communities.^1^ Nutrition is the cornerstone of the socio-economic development of a country. Malnutrition is not only the cause of mortality and morbidity but also leads to physical and mental impairment in children.^2^ Childhood malnutrition is a massive crisis caused by a combination of factors including inadequate food intake, childhood diseases, harmful childcare practices and low socio-economic status; all these contribute to poor health and millions of deaths annually.^3^ The United Nations Children’s Fund (UNICEF) describes malnutrition as a silent emergency that places a heavy burden on many families with low income.^4^ The World Health Organization (WHO) advocated for home-based health care to ensure improved accessibility to effective and efficient health care for communities in home settings. Home-based care also aims to improve the health and well-being of children and contributes to improving mortality and morbidity.^5^ Nutrition is increasingly recognised as a basic pillar for social and economic development; as such, reduction of infant and young child malnutrition is essential.^6^

Mid-upper arm circumference (MUAC) is useful for both screening of acute under-nutrition and for estimating the prevalence of malnutrition at a population level. The MUAC can be assessed with ease and this makes it suitable for nutritional screening during the height of an emergency, where time and skilled personnel are unavailable, and it can be performed during home visits by community caregivers (CCGs).^7^ The South African government has implemented various strategies to address the problem of malnutrition. One such strategy was community-based nutritional assessment, in which CCGs assess malnutrition through measuring MUAC using a tape in children under 5 years of age, for early identification of malnutrition and early referral to health facilities. The researcher observed that children present at the clinic with severe malnutrition, when it is expected that CCGs will have identified malnutrition early. For the period January 2014 through April 2015, 187 babies and children presented in primary health care clinics in North Area 6 of eThekwini district with severe malnutrition. Although a large percentage of children were malnourished, there was late detection and referral by the community health workers (CHWs).^8^ The aim of the study was to explore and describe the experiences of CCGs with the assessment of malnutrition in children under 5 years of age.

## Research aim and objective

The aim of the study was to explore and describe the experiences of CCGs on the assessment of malnutrition in children under 5 years of age.

## Method

### Study design

A cross-sectional, qualitative, exploratory, descriptive design was used to conduct the study.

### Setting

The study was conducted in eThekwini district in KwaZulu-Natal (KZN) province. eThekwini Municipality is divided into three subdistricts, namely North, South and West. The North is further subdivided into six areas; the study was conducted in North Area 6 of eThekwini district, which is 1 of 11 districts in KZN province, South Africa. The North subdistrict is home to approximately 1.5 million people, who comprise 31% of the population of eThekwini Municipality. This is the most populous subdistrict and 18% of the population live in this subdistrict. There are three community health centres in this subdistrict, with one district hospital. North Area 6 is at the boundary between eThekwini and iLembe districts. The total number of CCGs in North Area 6, both trained and untrained in nutritional assessment, was 180.

### Study population and sampling strategy

CCGs, previously known as CHWs, provide basic health care to communities they live in. Selection is a joint effort between community and local health structures. They are selected from the community, based on their interest in community work and good personal traits such as honesty, trustworthiness, willingness to serve the community and interest in community development, among others, and must be above 18 years of age and a permanent resident of the community. Supervisors must have done the same work for at least a year, must be able to read and write English and must have a track record of providing high quality care within the community.^10^ Their training includes mother and child health, where they do postnatal care, promotion of exclusive breastfeeding and growth monitoring; monitoring of adherence to treatment for mental health, tuberculosis (TB) and HIV and/or AIDS patients; and giving of health information on non-communicable and communicable diseases and nutrition. There were 78 CCGs trained in nutritional assessment in North Area 6. Thirteen purposively selected CCGs trained in community-based nutrition assessment and employed by both eThekwini municipality and Provincial Department of Health in the North subdistrict were included in the study:

- Inclusion criteria: CCGs trained in nutrition assessment and receiving a stipend.
- Exclusion criteria: CCGs not trained in nutrition assessment and those who are volunteers.

### Data collection

Data were collected using semi-structured, individual, face-to-face interviews in a private, quiet place by the researcher where CCGs worked in communities and lasted about 20–30 min. The interview guide contained a grand tour question: ‘what are your experiences with assessing malnutrition in children under 5 in the community using a MUAC measurement?’ This was followed by probing to elicit further details. Interviews were conducted in isiZulu, which is the home language spoken by all CCGs. The researcher is fluent in both English and isiZulu; no translation was required. Interviews were audio recorded and notes were made. The researcher transcribed the interviews verbatim; thereafter they were translated into English by a qualified language practitioner and back to isiZulu to ensure that richness of data was not lost during translation. Interviews were conducted once; however, a member check was conducted whereby the researcher returned to provide feedback to participants regarding their responses in the interviews to ensure that what the researcher captured was what participants meant.

A few interviews were conducted in the morning; most were conducted in the afternoon when CCGs were done for the day. Predetermined criteria were used to select these participants: CCGs who were trained in nutrition assessment and were receiving a stipend. The same interview guide was used to interview participants who worked in different types of households. In the member check the researcher returned to provide feedback to participants regarding the responses in the interviews to ensure a correct understanding of what responses meant.

### Data analysis

Data were analysed using content analysis, coded and labelled according to the created categories. Strategies used for trustworthiness were credibility through time and space triangulation, which was achieved by interviewing participants from different types of households in North Area 6. The researcher developed an audit trail where all original records of interviews and discussions were kept in a disc under lock and key for 5 years for dependability. For confirmability, the researcher supported interpreted data by using direct quotes from the recorded audit to eliminate subjectivity and bias from the study. Clear explanation of the limitations of the study were provided by giving clear descriptions of the study context, setting and research process to enable the reader to establish the transferability of results.

## Ethical consideration

Ethical approval was obtained from Durban University of Technology Institutional Research Committee (REC141/15); in addition, permission was obtained from the eThekwini Municipality Health Research Unit and the KwaZulu-Natal Department of Health. The ethical principles of beneficence, respect for human dignity and justice were upheld for participants.

## Results

### Theme 1: Training of community caregivers

Subthemes that emerged from this theme were as follows: inadequate training, scope beyond CCGs’ training and a lack of support and supervision (see Table 1). Participants perceived that these were the reasons for not being able to assess malnutrition in children under 5 years of age.

**TABLE 1:** Themes and subthemes.

Theme	Subtheme
Training of CCGs	Inadequate MUAC training Scope beyond the CCG’s level of training Lack of support and supervision
Remuneration	Stipend Contracts
Non-prioritisation of MUAC	Increased workload for the CCGs Shortage of CCGs in the community
Safety and security of CCGs on home visits	High level of alcohol consumption in the community Long walking distances for CCGs Perceived attitudes of community towards CCGs

MUAC, mid-upper arm circumference; CCGs, community caregivers.

#### Inadequate training

Participants had high levels of dissatisfaction with their training, which they considered inadequate. They felt that they lacked skill and knowledge in performing MUAC, as communities and nursing staff did not trust them. Participants stated:

‘The other day I observed the professional nurse measuring MUAC on the child’s arm and I discovered that I was measuring MUAC incorrectly. As CCGs, we need help from qualified health care professionals to polish our MUAC skills.’ (Participant 2: Female, 44 years, Grade10)

‘I performed MUAC on a child during a home visit and I did not refer the child – according to my observation the child was not malnourished; on the following week the child was taken to the clinic for other reasons and was found to have early signs of malnutrition.’ (Participant 9, Female, 25 years, Grade 11)

#### Scope beyond community caregivers’ training

Community caregivers felt that MUAC was beyond their level of training, as they mentioned that they did not understand everything during training because of the English language, which was the only language used for training. This theme was expressed as follows:

‘There was information that I did not understand in the training; if I asked my colleagues they did not answer me confidently and I felt this task was beyond our level, as the training was conducted in English.’ (Participant 1, male, 32 years, Grade12)

‘I felt that assessment of malnutrition in children under 5 years was a difficult task to understand in terms of the level of the CCGs. It was not supposed to be performed by the CCGs, as some of us have low levels of basic school education.’ (Participant 7, Female, 24 years, Grade 12)

#### Lack of support and supervision

Support and supervision were perceived as lacking; CCGs felt neglected without ongoing mentorship from skilled health care professionals, which could help to strengthen their performance in assessing malnutrition.

‘Since we started performing MUAC in communities, we were advised to report challenges to the clinics, but we are getting no support and supervision either from clinics or any other health department.’ (Participant 4, Female, 43 years, Grade 8)

‘The person who is our supervisor has her own area and home visits to do, so she does not have time to come to our areas to assist us. We have worked on our own since we came back from training.’ (Participant 2, Female, 44 years, Grade 10)

‘When we completed training, we were advised to report challenges to the clinics, who would allocate a health care professional to look after us, but we are getting no support, and no one is supervising us, either from the clinics or any other health department, who could experience what we are experiencing and help us.’ (Participant 4, Female, 4 years, Grade 8).

### Theme 2: Remuneration

All participants expressed that they were dissatisfied with the system of remuneration, as they did not know whether they would be paid at the end of the month. They were concerned that the stipend was too small, was paid at irregular intervals and sometimes not paid at all.

#### Stipend

‘I do not get additional money from the government for assessing malnutrition. This stipend is too little and it does not increase, but we get added work.’ (Participant 9, Female, 25 years, grade 11)

‘I cannot pay for my private studies from the stipend; it gets finished before I think of what to do with it, it is so little.’ (Participant 4, Female, 43 years, Grade 8)

#### Contracts

Delayed renewal of contracts was a negative experience for CCGS as some were unable to work while waiting to renew contracts because contracts are linked to receiving the stipend. Their patients were not receiving care during this time. This what they said:

‘The renewal of contracts is a nightmare; you cannot guarantee that your contract will be renewed until it is done. Mine was not renewed for 2 months and I did not get an explanation. It was renewed in the third month after everyone else’s was renewed.’ (Participant 2, Female, 44 years, Grade 10).

‘There was a time when we worked without pay for six months because our contracts had not been renewed and no one explained to us what was happening, what was the reason for not renewing the contracts.’ (Participant 5, Female, 40 years, Grade 6)

### Theme 3: Non-prioritisation of mid-upper arm circumference

#### Workload

Participants reported that they had other commitments in the community, which they felt were more important than MUAC because the children were not sick. This was expressed as follows:

‘I had four patients for daily home visits in my area and I had to supervise their TB and AIDS treatment in the morning afternoon. I did not perform MUAC on children until these patients were discharged.’ (Participant 4, Female, 43 years, Grade 8)

‘I had a very sick patient in my area that had no one to help her. Every day I did home visits only to her house because I needed to bathe, clean, cook and feed her; I could not do MUAC.’ (Participant 13, Female, 34 years, Grade 12)

#### Shortage of community caregivers

There was a shortage of CCGs in the community that made them unable to perform MUAC to their satisfaction. The CCGs expressed the problem as follows:

‘A colleague on my team was selected for nurse training and I was given her work load, and it was very hard, especially with TB defaulters; sometimes I ended up not assessing children.’ (Participant 9, Female, 25 years, Grade 11)

‘We have a number of home visits to do, but you find yourself doing more because there are areas that have no one, yet they need help.’ (Participant 1, Male, 32 years, Grade 12)

### Theme 4: Safety and security

Participants were concerned for their safety and security because they worked in dangerous areas with high rates of abuse of women and children in informal settlements. There was also a high level of alcohol consumption in communities and long walking distances, which exposed them to unsafe conditions.

#### Level of alcohol consumption

‘We did a home visit and we found the couple drunk and not looking after the three -year-old child; they began using abusive language towards us and we decided to leave.’ (Participant 4, Female, 43 years, Grade 8)

‘While waiting for a taxi under the tree, three drunk young men came and asked for our bags; fortunately, a vehicle stopped and the men in that vehicle assisted us.’ (Participant 8, Female, 58 years, Grade 7)

#### Long walking distances

Participants walked long distances to homes; some were hard to reach as no transport went to those areas. These long walking distances exposed them to unsafe conditions. They stated:

‘We did a home visit with my teammate and we did MUAC. The child had malnutrition; the mother told us that she will not be able to take her child to the clinic as it was far, and she did not have money, and it was very dangerous to walk. We were disappointed after we had walked such a distance to help her.’ (Participant 3, Female, 55 years, Grade 6)

‘Other areas were very far to reach; we needed to use transportation to reach those areas and sometimes there is no transport going to that area because it is too steep.’ (Participant 11, Female, 53 years, Grade 8)

#### Attitudes towards community caregivers

Participants reported that the community did not show appreciation towards the work they were doing. They mentioned that communities shut their doors when they saw the CCG approaching. This is what they said:

‘In one home visit the lady shut the door when she saw me approaching, and I could not continue doing MUAC on her children because she had shown that she did not have time for me.’ (Participant 10, Female, 30 years, Grade 10)

‘In another house they told me that the children’s parents were unavailable and shut the door on my face; they did not give me the opportunity to do MUAC.’ (Participant 5, Female, 40 years, Grade 6)

## Discussion

### Key findings

The CCGs were inadequately trained and were, therefore, not equipped to assess malnutrition, as they had not mastered the skill of doing MUAC and had not understood how to use some of the equipment, because of the language barrier. They lacked ongoing support and supervision in the field. Participants found themselves unable to assess malnutrition because of other work that took priority in the household. In some houses, they were not welcomed and were not given an opportunity to do their work of assessing malnutrition. The stipend was not regular and this was a concern to the CCGs. Their workloads increased with the introduction of new programmes without a reciprocal increase in the number of CCGs. Safety was not guaranteed as they walked long distances and community members consumed alcohol.

### Discussion of key findings

Growth monitoring and promotion of optimal nutrition are essential components of health care for all children. Monitoring a child’s nutrition status helps to confirm a child’s healthy growth and development. It also identifies early potential nutrition or health problems; hence it is critical to offer malnutrition assessment training to suitable candidates.^10^ The participants had high levels of dissatisfaction with their training, which was identified as inadequate. Providing nutrition training to implementers prior to implementation helps them to demonstrate higher levels of nutrition knowledge and counselling skills, which they lacked.^11^ Lack of understanding during the training process might have been because of the low level of basic education of some participants, as the training was conducted in English, which not all participants understood well. It is important that necessary and appropriate criteria be followed during the initial selection of CCGs and it should be facilitated by communities in a more participatory approach. However, that could have drawbacks because of the common tendencies of communities to select people related to community leaders, even if they are illiterate.^12^ The participants perceived the assessment of malnutrition in children under 5 years of age as beyond their level of training. They experienced many challenges in practice arising from not clearly understanding the training programmes. The participants lacked the skills and knowledge for malnutrition assessment regardless of training. This might have been because of the lack of ongoing supervision in the field. It is widely accepted that the quality of nutrition counselling is often poor, whether it be in growth monitoring and promotion programmes for improving complementary feeding or for community-based rehabilitation.^13^ CCGs were not mentored; they reported that they experienced technique difficulties when performing MUAC measurement. Intensive field supervision of CCGs with weekly contact sessions for mentoring might have increased CCGs’ nutritional assessment skills.^14^ The participants perceived that they should have been given extra incentives specifically for the assessment of malnutrition in children under 5 years of age. The participants reported that they were operating in highly challenging and stressful circumstances, yet their needs and priorities were ignored. Donors are willing to fund organisational events and activities but are reluctant to invest in staff salaries related to community health-based care activities.^15^ Contracts were not renewed when they were due and payment dates were also irregular; these problems caused great inconvenience for participants. Interruptions in the flow of funding from government sources to community home-based care workers via collaborations with non-profit organisations and the persistence of fragmented programme-specific approaches to service delivery instead of integrated, comprehensive approaches still exist.^16^ In 2016, the Department of Health proposed an extension of the contract periods for CCGs from the then 12 months to 24 months. This would enable the CCGs and supervisors to plan and stabilise their living conditions.^17^

The CCGs had multiple roles in the community, which compromised the assessment of malnutrition. The burden of HIV and/or AIDS and TB caused their workload to increase. They were directly observing treatment for patients on TB treatment, tracing treatment defaulters, giving Vitamin A drops to children and joining immunisation campaigns in the community. There is a need for further efforts by government to nurture CCGs so that they understand how young children within their communities need their assistance as a priority.^18^ There was a drastic shortage of CCGs in the community; CCGs who were selected for nurse training were not replaced; and their work areas were attended to by the remaining CCGs, whose numbers had been depleted. The more people that are reached, the more children will be saved and the closer we will come to winning the monumental fight against malnutrition and its life-long effects on children under 5 years.^19^ In the current study the findings indicated that participants were all concerned about their safety and security in the communities they worked. The participants reported that alcohol consumption was very high in the community and once people were under the influence of alcohol they used abusive language either towards each other in the presence of the CCGs or towards the CCGs. The participants perceived that those threatening situations in some communities contributed to MUAC not being performed in those areas. They walked long distances to reach communities and it was because of these long distances that the community members did not take their children to clinics after referral. The majority of the unemployed and most of the poor population reside in informal settlements and rural sites, which are known as hard-to-reach areas. This could be described as geographical unsafe or dangerous conditions.^20^

It is clear that the expression of confidence in CCGs is important to come from all role players. This might be the key to the acceptability of CCGs during home visits. Such confidence is related to training, skills and competencies of the CCGs, so it is important that CCGs are given the required skills for the implementation of the assessment of malnutrition in children under 5 years. A formal evaluation of the knowledge and skills of CCGs to undertake the assessment of malnutrition in children after completion of training is required, to ensure that CCGs have the skills to implement the programme effectively.

## Recommendations

Selection of CCGs for training should be based on their level of basic school education, from at least Grade 10 upwards, because the training is conducted in English. This might assist with the language barrier. On the other hand, consideration should be given to having facilitators who speak the same language as the trainees.

Ongoing support and supervision should be given to CCGs to ensure that their skills are accurate and are not lost.

CCGs do more than MUAC in the community; it is important to meet regularly to get feedback from them on the work that they do so as not to compromise checking child nutrition when they have to prioritise other duties such as assisting helpless patients.

Supervisors must be available to assist CCGs and identify training gaps. Ongoing training (in service) should be implemented to ensure that CCGs retain the skill of performing MUAC. Individual attention may assist those who did not understand during the initial training.

Community meetings must be held to discuss the deployment of CCGs so that they are accepted and treated with respect. This might also improve their safety. Communities need to be taught the importance of child nutrition so that they accept and appreciate CCGs when they come to their homes to perform this function and will allow the CCGs to work without fear or hindrances.

## Study limitations

The study was conducted only on CCGs who were trained in assessing malnutrition in children under 5 years using MUAC. CCGs not trained in malnutrition assessment and volunteers were excluded from the study. The study was conducted in the North subdistrict; other areas were excluded.

## Conclusion

Malnutrition assessment in children under 5 years remains problematic in communities. Selection of CCGs is not structured, which could pose a challenge during the training, which is conducted in English, a language that most participants in this study did not understand well. There was no support and supervision, which caused a lack of accurate assessment of malnutrition in children in the community. Furthermore, there was dissatisfaction with how they were barred from doing their job, as it was not viewed as important by communities. CCGs work under hostile conditions, when the communities they serve should protect them. CCGs are an important part of the health system. If these challenges were rectified, it would improve the manner in which CCGs do their job, thus contributing to the health and well-being of communities, especially babies and children.
